# MicroRNAs and Mammarenaviruses: Modulating Cellular Metabolism

**DOI:** 10.3390/cells9112525

**Published:** 2020-11-23

**Authors:** Jorlan Fernandes, Renan Lyra Miranda, Elba Regina Sampaio de Lemos, Alexandro Guterres

**Affiliations:** 1 Hantaviruses and Rickettsiosis Laboratory, Instituto Oswaldo Cruz, Fundação Oswaldo Cruz, Rio de Janeiro 21040-900, Brazil; jorlan@ioc.fiocruz.br; 2 Neurochemistry Interactions Laboratory, Universidade Federal Fluminense, Niterói 24020-150, Brazil; renan_miranda@id.uff.br

**Keywords:** microRNAs, mammarenaviruses, cellular metabolism, amino acid metabolism, metabolism of cofactors and vitamins

## Abstract

Mammarenaviruses are a diverse genus of emerging viruses that include several causative agents of severe viral hemorrhagic fevers with high mortality in humans. Although these viruses share many similarities, important differences with regard to pathogenicity, type of immune response, and molecular mechanisms during virus infection are different between and within New World and Old World viral infections. Viruses rely exclusively on the host cellular machinery to translate their genome, and therefore to replicate and propagate. miRNAs are the crucial factor in diverse biological processes such as antiviral defense, oncogenesis, and cell development. The viral infection can exert a profound impact on the cellular miRNA expression profile, and numerous RNA viruses have been reported to interact directly with cellular miRNAs and/or to use these miRNAs to augment their replication potential. Our present study indicates that mammarenavirus infection induces metabolic reprogramming of host cells, probably manipulating cellular microRNAs. A number of metabolic pathways, including valine, leucine, and isoleucine biosynthesis, d-Glutamine and d-glutamate metabolism, thiamine metabolism, and pools of several amino acids were impacted by the predicted miRNAs that would no longer regulate these pathways. A deeper understanding of mechanisms by which mammarenaviruses handle these signaling pathways is critical for understanding the virus/host interactions and potential diagnostic and therapeutic targets, through the inhibition of specific pathologic metabolic pathways.

## 1. Introduction

Mammarenaviruses (*Bunyavirales*: *Arenaviridae*: *Mammarenavirus*) are enveloped bi-segmented ambisense RNA viruses and each segment encodes for two oppositely oriented non-overlapping reading frames separated by an intergenic region, a small (S) segment that encodes the envelope glycoprotein precursor (GPC) and the nucleoprotein (NP) and a large (L) segment coding for the matrix protein (Z) and the RNA-dependent RNA polymerase (RdRp) [1,2]. Based on their genomic features and antigenic properties, mammarenaviruses are historically classified into two large monophyletic clades—the Old World (OW) and New World (NW) groups, both containing important zoonotic pathogens affecting humans. NW mammarenavirus, or Tacaribe serocomplex, includes viruses indigenous to the Americas and can be further divided into Clades A, B, C, and D while the OW or Lassa-lymphocytic choriomeningitis virus (LCMV) serocomplex includes viruses from Africa and, recently, some viruses isolated in Asia [1,3,4].

Some of these emerging, zoonotic viruses are pathogens of major clinical importance to humans, as (i) Lassa virus (OW) is endemic in West Africa and is estimated to cause up to 300,000 infections each year, (ii) Junín (Clade B NW) that periodically cause hemorrhagic fever (HF) outbreaks in Argentina, and (iii) LCMV (OW) which is distributed worldwide and is a neglected human pathogen of clinical significance, to immunocompromised individuals and pregnant women, due to teratogenicity [5,6,7]. Although these two groups share many similarities, important differences with regard to pathogenicity, type of immune response, and molecular mechanisms during virus infection recovery and clearance of the virus is different between and within NW and OW infections. For example, whilst both the OW Lassa virus (LASV) and Junín virus (JUNV) can cause disruption of the vascular endothelium, which is an important pathological feature, the immune responses for LASV infection results in an overall generalized immune suppression, and patients infected with JUNV seem to develop a cytokine storm [8,9]. Understanding the potential differences on a cellular level could aid the development of new vaccines and treatment strategies against these deadly viral infections.

MicroRNAs (miRNAs) are the smallest endogenous regulatory non-coding RNAs that play a central role in cell differentiation, proliferation, and survival by binding to complementary target mRNAs leading to translational inhibition or degradation. miRNAs are the crucial factor in diverse biological processes such as antiviral defense, oncogenesis, and cell development [10]. There are complex interaction networks between long non-coding RNAs (lncRNAs), viral genome, and miRNAs. While certain miRNAs can regulated the stability and half-life of lncRNAs and viral genome, lncRNAs and viral genome can also compete for miRNAs acting as miRNA sponges and epigenetic modifications [11,12]. The miRNA-binding sites within viral genomes are mostly located in the 5′ and 3′ non-translated regions (NTRs) but have recently been found in the coding regions of viral proteins [12,13,14]. Viral genomes and gene transcripts affect with host gene expression exploiting passive mechanisms to deregulate host miRNA activity. Various RNA viruses mimic or block the binding between a host miRNA and its target transcript, a phenomenon mediated by the miRNA seed site at the 5′ end of miRNA [12,15]. Based on bioinformatics, we predicted the potential miRNAs, their target genes, and related signaling pathways. To the best of our knowledge, this is the first study to systemically analyze and predict the potential miRNAs and target genes for *Mammarenavirus*. Our research could help to further assess the roles of miRNAs in virus–host interactions, highlighting potential targets during infection, leading to a better understanding of the metabolic alterations required for the replication of each virus that may lead to novel therapeutic approaches through targeted inhibition of specific cellular metabolic pathways.

## 2. Materials and Methods

The genomic sequences used in the study were all retrieved from the GenBank^®^ database of NCBI (http://www.ncbi.nlm.nih.gov/nuccore/), including all species of the genus *Mammarenavirus* officially recognized by the International Committee on Taxonomy of Viruses (ICTV) (https://talk.ictvonline.org/taxonomy/). Herein, for a more clear presentation of our results, we considered Clade D New World mammarenaviruses (formally known as Clade A recombinant) as members of Clade A, as well as Xapuri virus that is associated with Clades B, and C was didactically classified here as belonging to Clade B NW mammarenaviruses.

Currently, the human genome contains 2654 mature sequences of microRNAs identified in the miRBase database (http://www.mirbase.org). We used BLAST with the Geneious R11.1 software (https://www.geneious.com) to search for miRNAs that interact with the mammarenavirus genome with a perfect alignment of 11 nucleotides encompassing the 8 mer seed region, important specific gene silencing motifs, and regions from the viral RNA [16]. We also sought interactions of miRNAs with transcribed mRNA for the viral proteins and antigenomic viral RNA that is produced during the replication in the *Mammarenavirus* genus.

For microRNA bioinformatic target prediction, we used miRabel, a tool that shows significantly better predictions than other important algorithms such as MBSTAR, miRWalk, ExprTarget, and miRMap [17]. This tool adds all human results of four important prediction algorithms, miRanda [18], PITA [19], SVMicrO [20], and TargetScan [21]. Each of them uses different and complementary features with interactions such as seed match, free energy, site accessibility, and target-site abundance. The miRabel contains data for 2587 human miRNAs which have target mRNAs, 19,799 genes and 275 pathways, representing more than 14.7 million predicted interactions from which 351,298 are experimentally established. These experimentally validated interactions were annotated using miRTarBase [22] and miRecords [23], whereas 5′UTR and CDS predictions were identified with the miRWalk database [24]. miRabel uses genes and pathways information as well as their relationships recovered from the KEGG database [25] while miRNA data were from miRbase (release 22.1) [26] and linked with miRNA target predictions.

miRabel produce pathways linked to the resulting interactions that can be retrieved and ranked according to the proportion of its interactions regulated by a given microRNA. Moreover, for each pathway, the number of validated interactions for this miRNA is also indicated [17]. To result in a robust prediction, we consider that the pathways that had 25% of proportion of its interactions are regulated by a given microRNA.

The data of mammarenaviruses–miRNA interaction and miRabel results were integrated using the python library pandas [27]. The resulting dataset was used to generate heatmaps to visualize the data with the python library seaborn. We used KEGG pathway classification and its hierarchy relation plotted against all mammarenaviruses. The scale demonstrates how many miRNA–pathway relationships were found for each virus species. If a miRNA targeted multiple pathways that were under the same higher order classification and if multiple miRNA targeted the same pathway, each time would count as a miRNA–pathway relationship in order to represent the possible relevance of miRNAs in that pathway. We started showing higher order pathway classification and subdivided it for better visualization. All procedures were performed using python programming language version 3.6.

## 3. Results

### 3.1. MicroRNAs

We retrieved a total of 39 genome sequences from GenBank^®^: 20 NW species, divided into Clade A (8), Clade B (10), and Clade C (2) viruses and 19 OW species. Among the 2654 mature miRNAs identified in the miRBase database, we found that 566 miRNAs can bind to certain regions of the mammarenavirus genome in a total of 755 binding-sites (Supplementary Table S1). We found 316 miRNAs (386 binding-sites) for NW mammarenavirus: 131 of Clade A species (147), 169 Clade B (194), and 45 for Clade C (45). Some of these miRNAs bind to more than one clade, totaling 345. Of these, 231 miRNAs (277) strongly bind in the L segment and 97 to the S segment (109). In total, 313 miRNAs (369) were able to bind in the genome of OW mammarenaviruses: 213 for the L segment (244) and 109 to the S segment (125) (Table 1). The average amount of miRNAs binding-sites by specie was nineteen, with Loie River virus (OW) presenting the lowest amount of predicted miRNAs (8) and Cupixi virus (Clade B NW) the highest (30).

Among the 521 binding-sites found within the L segment, 208 were found in the RNA-dependent RNA polymerase (RdRp) region for OW mammarenaviruses and 259 for NW (129 for Clade B, 100 Clade A, and 30 Clade C). We found 27 binding-sites in the Z protein (Z), being 17 for OW and 10 for NW (4 Clade A, 4 Clade B, and 2 Clade C). We identified 19 binding-sites for the noncoding intergenic region of the L segment (L-IGR), with 13 for OW mammarenaviruses, 5 for Clade B, and 1 for Clade A. We have not identified any interaction to L-IGR of the Clade C. Regarding 5′UTR and 3′UTR untranslated region, we found four binding-sites to each region. In total, 233 binding-sites were found within the S segment, being 114 for glycoprotein precursor (GPC), 109 for nucleoprotein (NP), 9 for noncoding intergenic region of the S segment (S-IGR), and 1 bind in the 3′UTR untranslated region. Further information can be found in Supplementary Table S1, where the region with which each miRNA interacts is informed.

Fifty-two miRNAs were found binding to more than one virus in the same genomic segment. For example, the hsa-miR-2052 can bind to the S segment of Allpahuayo virus, Bear Canoyn virus, Pichinde virus, and Pirital virus, all belonging to Clade A NW (Table 2). Another interesting example is the hsa-miR-3120-3p binding to L segment of Lujo virus, Lunk virus, Merino Walk virus, and Mopeia virus, all from OW. The complete table with all miRNAs is available in Supplementary Table S2.

Thirty-four miRNAs can bind to different regions of L and S segments. For example, hsa-miR-122b-3p can bind to two different positions of Aporé virus L segment, Flexal virus, Machupo virus, Paraná virus, and also to Alxa virus S segment. Another interesting miRNA was miRNA hsa-miR-9-5p that can bind in Pichindé virus L segment, Ryukyu virus, Mobala virus, and to Tacaribe virus S segment (Table 3). The complete table with all miRNAs is available in Supplementary Table S3.

Ten miRNAs were predicted to bind different regions of the same virus, especially the miRNA has-miR-8485 binding in three different positions of Ryukyu virus L segment and five positions of Lunk virus L segment, both belonging to OW mammarenaviruses group (Table 4).

### 3.2. miRNAs and Target Metabolic/Cellular Pathways

To further understand the biological implications of miRNAs binding to the mammarenavirus genome, miRabel was used to predict genes targeted by these miRNAs (data not shown). After genes prediction analysis, we performed pathway enrichment analysis of miRNAs target genes based on the KEGG database using miRabel and generated a report of the pathway mapping. Our analysis found a total of 75 pathways (Supplementary Figure S1).

**Figure 1:**
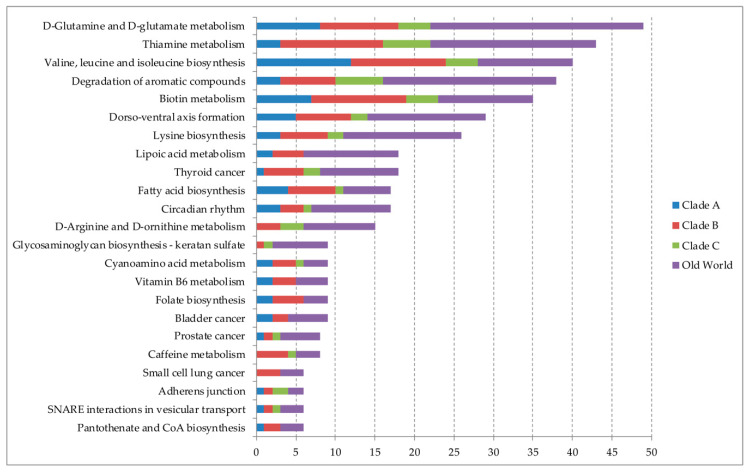
Top 20 main signaling pathways predicted to be regulated by microRNAs targeting mammarenavirus genome. The representative proportion of each pathway individually for the four clades (Clade A, B, C, and Old World) is shown. The graphic scale is represented by the number of microRNAs that were found interacting with each pathway. The larger the scale, the greater the number of microRNAs found that are regulating a specific pathway.

Overall, d-Glutamine and d-glutamate metabolism, thiamine metabolism, and valine, leucine, and isoleucine biosynthesis were the main signaling pathways predicted to be regulated by miRNAs (Figure 1). A total of 23 pathways were predicted to Clade A where valine, leucine, and isoleucine biosynthesis, d-Glutamine and d-glutamate metabolism, and biotin metabolism were the main signaling pathways; 55 pathways for Clade B, the top three being thiamine metabolism, biotin metabolism, and valine, leucine, and isoleucine biosynthesis, both in second place and d-Glutamine and d-glutamate metabolism in third place; and 29 for Clade C viruses, where degradation of aromatic compounds and thiamine metabolism were the most common followed by d-Glutamine and d-glutamate metabolism, valine, leucine, and isoleucine biosynthesis, and biotin metabolism, all in second. For miRNAs binding to Old World mammarenavirus, we found a total of 62 predicted pathways, d-Glutamine and d-glutamate metabolism, degradation of aromatic compounds, and thiamine metabolism were main ones (Supplementary Figure S2).

The miRabel tool through the KEGG pathway analysis demonstrated that the targets of miRNAs were more associated with amino acid metabolism and metabolism of cofactors and vitamins. d-Glutamine and d-glutamate metabolism pathway was regulated by miRNAs for a total of 49 times (miRNAs encountered in two or more clades were considered multiple times), 8 miRNAs that were found for Clade A, 10 for Clade B, 4 for clade C, and 27 for OW mammarenaviruses, including miRNAs that were found in more than one group of viruses. The thiamine metabolism was targeted 43 times, 3 miRNAs for Clade A, 13 for clade B, 6 for Clade C, and 21 for OW viruses. The results showed a total of 40 miRNAs interactions with the pathway with the valine, leucine, and isoleucine biosynthesis, 12 for Clade A, 12 for Clade B, 4 for Clade C, and 12 for OW viruses.

**Figure 2:**
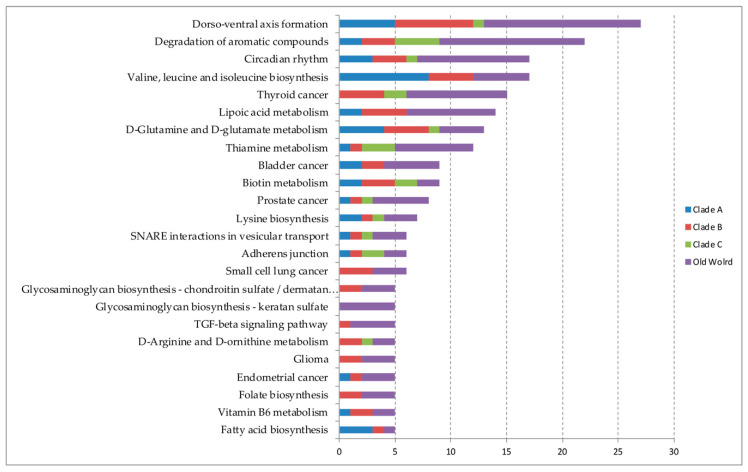
Top 20 main signaling pathways predicted to be regulated by microRNAs biding to mammarenavirus genome that had at least one target validated experimentally in proportion for each of the four clades (Clade A, B, C, and Old World viruses). The graphic scale is represented by the number of microRNAs that were found interacting with each pathway. The larger the scale, the greater the number of microRNAs found that are regulating the pathway.

When considering pathways that have at least one experimentally validated interaction for miRNAs, we found a total of 69 pathways out of the 75 initials (Supplementary Figure S3). Dorso-ventral axis formation, degradation of aromatic compounds, and circadian rhythm were the main validated signaling pathways predicted to be regulated by miRNAs (Figure 2). According to each mammarenavirus group, 20 pathways were found in Clade A viruses and valine, leucine, and isoleucine biosynthesis, dorso-ventral axis formation, and d-Glutamine and D-glutamate metabolism were the three main predicted pathways; for Clade B a total of 49 pathways were found with dorso-ventral axis formation first of all, and d-Glutamine and d-glutamate metabolism, Lipoic acid metabolism, valine, leucine, and isoleucine biosynthesis, and Thyroid cancer, equally represented, stand out in proportion. Twenty-two were found for Clade C with degradation of aromatic compounds and thiamine metabolism, biotin metabolism, adherens junction, long-term depression, and thyroid cancer, highlighted in miRNA proportion by pathway. For those miRNAs that were predicted in OW mammarenaviruses, we found a total of 59 pathways, especially dorso-ventral axis formation, degradation of aromatic compounds, and circadian rhythm.

Among all predicted pathways, only seven showed no experimentally validated interactions for the set of miRNAs: caffeine metabolism; phenylalanine, tyrosine, and tryptophan biosynthesis; primary bile acid biosynthesis; proximal tubule bicarbonate reclamation; shigellosis; and synthesis and degradation of ketone bodies.

### 3.3. Mammarenavirus Species, miRNAs, and Target Metabolic/Cellular Pathways

**Figure 3:**
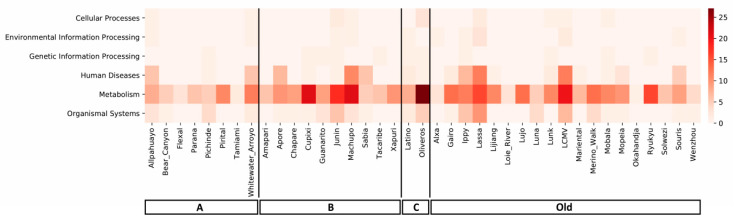
Heatmap of predicted miRNA–pathway group interactions for each mammarenavirus. Scale demonstrates how many times a pathway that belonged to a KEGG pathway group was targeted by a miRNA (if a miRNA targeted more than one pathway in the same group it was counted as many times).

We used KEGG pathway classification in order to predict biological relevance of each miRNA found for the different mammarenavirus included in this study. Target genes of miRNAs found were associated with six branches of KEGG pathways: cellular processes, environmental information processing, genetic information processing, human disease, metabolism and, organismal systems. Notably, target genes of miRNAs found are more frequently related to pathways that belong to the metabolism branch, followed by human diseases and organismal systems (Figure 3). Strikingly, the set of miRNAs found for Junín virus, Machupo virus, Lassa virus, and Lymphocytic choriomeningitis virus (LCMV) act strongly in these three branches of KEGG pathways.

**Figure 4:**
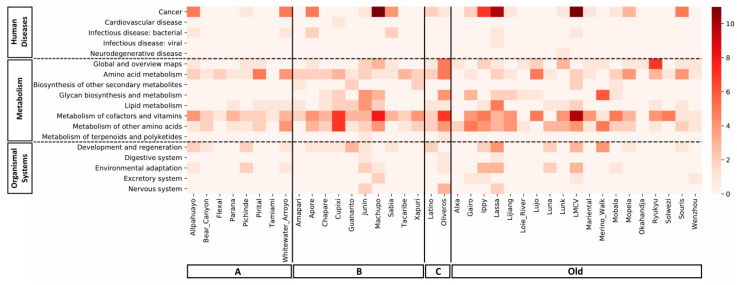
Heatmap of predicted miRNA–pathway subgroup interactions for each mammarenavirus. Scale demonstrates how many times a pathway that belonged to a KEGG pathway subgroup was targeted by a miRNA (if a miRNA targeted more than one pathway in the same subgroup it was counted as many times).

Within the KEGG metabolism classification, the predicted areas to be more affected by mammarenavirus infection are metabolism of cofactor and vitamins, metabolism of other amino acids, and amino acid metabolism. Pathways related to cancer studies are highlighted within human diseases group. For organismal systems, interactions occurs more frequently in the development and regeneration pathway. We also observed that the miRNAs found for the Junín, Machupo, Oliveros, Lassa, and Lymphocytic choriomeningitis viral genome were the ones with the greatest impact on different pathways (Figure 4). For example, miRNAs that target certain regions of the Lassa genome act in six of the eight metabolism pathways, also acting in four of the five organismal systems pathways, and in three of the five of human diseases pathways.

Individually evaluating each virus, we found different sets of mapped pathways. For example, for Pirital virus, miRNAs are mainly associated with the valine, leucine, and isoleucine biosynthesis. Olivero virus degradation of aromatic compounds followed by biotin metabolism were the main predicted pathways. MicroRNAs found for Lujo virus were mainly related to thiamine metabolism (Supplementary Figure S4).

## 4. Discussion

MicroRNAs are noncoding RNAs which downregulate a large number of target mRNAs and modulate cell activity [10]. MicroRNAs functions under normal physiological conditions might be integrated into multilayered control circuits ensuring proper development and cellular homeostasis. However, the dysregulation of miRNA expression or function in response to intrinsic factors (genetic or epigenetic) or extrinsic factors (environmental cues or stress, such as viral infection) may contribute to aberrant gene expression patterns underlying abnormal developmental patterning or metabolic dysfunction [28,29]. Viral genomes could have evolved to directly interact with host miRNAs to facilitate certain steps of their replication and progression. The cellular miRNA composition in infected cells is likely to indirectly affect viruses, because many pathways that promote or limit viral replication or the survival of infected cells are likely to be regulated by cellular miRNAs. In either case, viruses may therefore gain an advantage by reshaping the cellular miRNAs availability [12,15,30,31].

MiRNAs are characterized by variable expression in cells and tissues, which is influenced by the molecular cell environment. Some different miRNAs and miRNA families are predominantly expressed in certain tissues. However, the majority of miRNAs (>80%) is not specific for single tissues [32]. A great example is miR-122 that is highly abundant in liver, with over 60,000 copies in hepatocyte cells [33]. The impact of miR-122 binding on the hepatitis C virus (HCV) genome is critical for viral replication. Upon binding of miR-122, HCV genome translation and initiation of replication are increased by stabilizing and protecting the uncapped HCV RNA genome from degradation [34,35]. MiR-122 in vivo knockout studies have revealed that reduced miR-122 expression in hepatocellular carcinoma correlates with metastasis and poor prognosis [36,37]. These discoveries provide insight into the importance of the roles of miRNAs in maintaining normal cellular function and how disruptions in miRNA expression profiles may heavily impact the development, differentiation, and control of growth leading to diseases.

In this article, we predicted several cellular miRNAs potentially regulated by mammarenavirus via direct binding to viral RNAs. Aside from virus specific binding sites, we also predicted cellular pathways that could be dysregulated during viral infection. A large intersection of predicted miRNA sets was found on miRNAs targeting cell metabolism for all mammarenavirus groups, including those considered as human pathogens. As known, viruses are entirely dependent on host metabolism to support its replication, and virally infected cells seem to require complex metabolic alterations in order to deal with the high anabolic demands essential during viral replication [38,39,40].

However, there are highly exclusive patterns of virus-induced remodeling of host cell metabolic machineries, and the mode of cell manipulation appears to be different between RNA and DNA viruses [40,41]. Metabolic studies were successfully applied to a diversity of virus infections in mammals, insects, and plants, besides chronic and acutely infected cell cultures. In all cases, important changes in host cellular metabolism were observed, allowing the identification of specific metabolites and pathways involved in viral infection, and potential cross-talk with the immune system and virus pathogenesis [42,43,44,45,46,47,48]. We identified that d-Glutamine and d-glutamate metabolism, thiamine metabolism, and valine, leucine, and isoleucine biosynthesis seems to be the most affected pathway during mammarenavirus infection. As previous studies have shown for other viral families, mammarenaviruses also could control host-cell metabolism via pos-transcriptional regulations to cope with the pace of the corresponding replication cycles [38,49].

Glutamate metabolism plays a vital role in biosynthesis of nucleic acids and proteins [50]. Glutamine is the most abundant and versatile amino acid in the body and is of fundamental importance to intermediary metabolism, interorgan nitrogen exchange via ammonia (NH3) transport between tissues, and pH homeostasis [51]. It is believed that the viral infection induces glutamine uptake and that glutaminolysis is required to generate cellular energy during infection, allowing the survival of infected cells [52,53]. In fact, LCMV infection of mice was found to change significantly the concentration of α-ketoglutarate, in a concerted fashion, following a response and recovery pattern [54]. The α-ketoglutarate is a product of a set of metabolic reactions that degrade glutamine (glutaminolysis), allowing it to be used anaplerotically to form the intermediates of the tricarboxylic acid cycle (Kreb’s cycle) supporting oxidative phosphorylation [55]. The plasma concentrations followed mice response and recovery dynamics, decreasing at day 3, reaching a minimum concentration at day 7, when viral infection peaks, and returning to the initial concentration at day 14 [54]. These results were also correlated to immune response, including the rise and fall of natural killer cell populations, serum soluble TNF receptor concentration, and viral clearance, providing potential miRNA-mediated pathway targets for molecular diagnostics or therapeutics of mammarenavirus infection and immune response.

Similar results have been reposted, where glutamine is important to support HIV-1 replication, when comparing HIV-1 infected with uninfected activated primary human CD4^+^ T cells. Glutamine concentrations are elevated in HIV-1-infected cells, implying that HIV-1 infection leads to considerable changes in the cellular glutamine metabolism [56]. In another study using metabolomics, researchers showed that the latently infected cells with Kaposi’s Sarcoma-associated herpesvirus (KSHV) have higher levels of glutamine as compared to their mock counterparts [48]. Lysine is another amino acid that has been shown to play an important role on viral infections. It is essential for the replication of viruses and progression of infections [57,58,59]. The extra- and intracellular concentrations of l-lysine amino acid play a limiting role in the synthesis of the virus proteins and in transcription initiation of the retrovirus life cycle, and the deficiency of this essential nutritional element can reduce viral load [60]. Studies have demonstrated serious changes in plasmatic concentrations of l-lysine in HIV-infected patients, where it was found that plasma concentrations of l-lysine were negatively correlated with HIV-1 RNA levels and inversely with CD4 lymphocytes count. Therefore, an excess of l-lysine concentration leads to active HIV replication and reduces the concentration of plasma amino acid [59,61].

In a recent review, Keshavarz and colleagues, based on available metabolomic studies, argued that influenza virus infection can affect with cellular metabolic pathways either directly or indirectly via stimulation of immune system mediators [62]. Curiously, through enhancing the activity of the mTORC1 complex, the influenza virus strengthens numerous metabolic pathways, including glycolysis, glutaminolysis, pentose phosphate, and fatty acid synthesis, to provide more ATP and structural materials for viral replication [62,63,64]. Mammalian target of rapamycin (mTOR) is a conserved serine/threonine kinase that plays a critical role in the control of cellular growth and metabolism [65]. Mapping of the mTOR signaling pathways has revealed that mTOR controls biomass accumulation and metabolism by modulating key cellular processes, including protein synthesis and autophagy [66]. Some important studies have revealed interplays between miRNAs and mTOR pathway during cancer development. These interactions appear to provide a fine-tuning of various cellular functions and contribute qualitatively to the behavior of cancer [67,68]. Furthermore, excessive upregulation of mTOR complex 1 (mTORC1) protein complex pathway could be detrimental because it leads to enhanced production of particular miRNAs [69].

Vital metabolic pathways of host cells are one of the most widely used mechanisms targeted by viruses, resulting in extensive modifications. Some studies have revealed that many different human viruses, such as dengue [70], cytomegalovirus [44,71], and rubella [72], can strongly affect host cell glycolysis, lipid metabolism, and glutaminolysis. Cheng and collaborators demonstrated that enterovirus 71-infected Vero cells had significant changes in glutathione and its related metabolites, and several amino acids, such as glutamate and aspartate. Furthermore, they associated the presence of glutamine in culture medium with an increase in viral replication, and a dimethyl α-ketoglutarate treatment partially mimicked the effect of glutamine supplementation [73].

The small molecule profile of serum from Lassa fever-infected patients indicated a physiological dysregulation affecting pathways mediating blood coagulation, and lipid, amino acid, nucleic acid metabolism changing the levels of numerous metabolites in the circulation [74]. Serum lipids were the most frequently identified molecular class and also the most frequently identified as decreased in fatal Lassa fever and LCMV infected mice as well as proteolytic breakdown products as the dipeptides γ-glutamyl-Valine, γ-glutamyl-Leucine, and prolyl-hydroxyproline, which were present in lower amounts over the course of LCMV infection. Suggesting possible changes in the concentration or activity of enzymes, Gamma glutamyl transferase transfers the gammaglutamyl moiety to various amino acids during viral immune response [54,74]. In this regard, our study predicted several miRNA related to amino acid and fatty acid biosynthesis and metabolic pathways accounting as a potential mammarenavirus strategy to subvert the cell metabolism to its benefits, increasing catabolism of unnecessary compounds and favoring synthesis or suppressing metabolism of those necessary for replication and evasion of immune system.

Biotin metabolism was among the most frequent pathways regulated by miRNAs that target mammarenavirus genome. Biotin may affect transcription of genes, biotinylation of proteins in cells, cellular growth, proliferation, and differentiation. The vitamin acts as a co-factor for five carboxylases that are critical for fatty acid, glucose, and amino acid metabolism [75,76]. Biotin deficiency is associated with various diseases, and vitamin deficient mice display enhanced inflammation. Moreover, abnormal cellular growth and differentiation is the underlying cause of fetal malformations [77,78] and impaired immune function observed in biotin-deficient animals [79]. The predicted miRNAs can play an important role in congenital LCMV infection that are marked by permanent, lifelong neurologic deficits, the most common are macrocephaly (secondary to hydrocephalus), and microcephaly [80,81]. Biotin was also shown to be important for the activity of human natural killer lymphocytes, for the generation of cytotoxic T lymphocytes, and for the maturation and responsiveness of immune cells [75,82,83].

One important pathway predicted to be associated with miRNAs targeting mammarenavirus genome was the circadian rhythm which is an internal biological clock that enables to sustain an approximately 24-h rhythm in the absence of environmental cues. The circadian clock system is a main regulatory factor for nearly all physiological activities and its disorder has severe consequences on human health [84,85]. Interestingly, Miller and colleagues performed a study where they infected mice with LCMV and measured plasma corticosterone and cytosolic glucocorticoid receptor (GR) binding at multiple time points throughout the day and throughout infection. Despite a vigorous immune response to LCMV, the infection was associated with minimal and transient increases in corticosterone secretion. However, significant decreases in cytosolic GR were detected in immune tissues. Receptor decreases were characterized by a significant reduction of GR binding during the diurnal rise in corticosterone in the spleen and thymus of infected but not uninfected animals on days 5–10 post infection. In addition, in the morning on these days, GR binding in the spleen of infected mice was decreased compared to uninfected control mice [86]. This indicates effects of LCMV infection in the circadian rhythm with interactions between the neuroendocrine and immune systems modifying at the level of the GR the context of an ongoing immune response during a viral infection.

Similar results were found by Edgar and colleagues in infected wild-type mice with a strain of herpes virus and influenza A at different phases of the animals’ circadian clocks. These researchers demonstrated that the time of day of virus infection has an important impact on disease progression, in cellular models as well as in animals. In addition, they observed that clock disruption leads to increased virus replication and dissemination, indicating that severity of acute infections is influenced by circadian timekeeping [87]. Since there is a possibility of circadian rhythms affecting the immune response in the animals, the timing of vaccination should also be important. Indeed, timing of influenza vaccine administration has been shown to be a determinant in the systemic immunization response in people over 65 years of age. In a cluster-randomized trial design, Long and collaborators examined whether manipulating the time of day an older adult received their influenza vaccination would have an effect on the magnitude of the antibody response at one month. Results showed that antibody responses to two of three influenza strains were higher when the vaccination was given in the morning [88].

The identification of the molecular determinants underlying the distinct virulence of pathogenic and nonpathogenic arenaviruses is still a question of great importance for epidemiology and public health [89]. In our study, there was no clear difference in total miRNAs found with the potential to bind to pathogenic and nonpathogenic mammarenavirus genomes, which could be explained by the close genetic relationship of nonpathogenic arenaviruses with their highly pathogenic cousins [1,89,90]. Although the role of miRNA in mammarenavirus pathogenesis is not clear, its role in regulating important molecular pathways during infection cannot be ruled out as an important mechanism to be further investigated. In addition, many studies have suggested that a multifactor pathway could be involved in the different outcomes from arenaviral infection including a relatively low number of adaptive mutations, as well as the incapacity to bind to human receptors and different immune responses elicited by these viruses [91,92,93].

Interaction between host and pathogen has a profound effect on the outcome of an infection. Knowledge about host–pathogen interactions is critical for understanding the pathogenesis of infection. However, it generally overshadows knowledge of metabolic cross-talk between host and pathogen [43,94]. At a cellular level, host and pathogen share similar nutritional substrates generating common metabolic products. The host depends upon nutritional substrates to support its immune responses against the pathogen, while the pathogen is also highly dependent on nutritional substrates being unable to synthesize some or all substrates for its replication [49,94,95]. Post-transcriptional regulation of gene expression plays a pivotal role in various gene regulatory networks including but not limited to metabolism, embryogenesis, and immune responses. Recently, the role of post-transcriptional gene regulation during pathogenic infections and host immune responses has become increasingly important. The role of cellular miRNAs is crucial when they are proviral, or when a longer, persistent infection is established. miRNAs can be used as an entry gate into regulatory networks that could be explored to find new unconventional therapeutic targets.

## 5. Conclusions

Our present study indicates that mammarenavirus infection induces metabolic reprogramming of host cells, probably manipulating cellular microRNAs. A number of metabolic pathways, including valine, leucine, and isoleucine biosynthesis, d-Glutamine and d-glutamate metabolism, thiamine metabolism, and pools of several amino acids were impacted by the predicted miRNAs that would no longer regulate these pathways. The change caused by the effect of the mammarenavirus genome acting as a sponge to miRNAs probably allows activation of anabolic pathways necessary for the production of the viral nucleic acids, capsids, and eventually membrane envelopes. While some studies have demonstrated that viruses reprogram cell metabolism and rely on metabolic changes for optimal virus replication, significant work remains to determine as viral genome interact with host cell machinery to induce such alterations and characterize whether the same metabolic perturbations occur during infection. As well as other viruses, the mammarenavirus has likely evolved to modify the host metabolism for multiple purposes, facilitating viral replication and potentially also reflect antiviral defense mechanisms. The study of metabolism, including metabolomics approach, should not be restricted to energy supply and biosynthetic materials, but should also extend to a better understanding of its role during viral infection and pathogenesis. A deeper understanding of mechanisms by which mammarenaviruses handle these signaling pathways is critical for better understanding the virus/host interactions and potential targets for diagnostic and therapeutic, through inhibition of the specific pathologic metabolic pathways.

## Figures and Tables

**Table 1:** Total miRNAs found with the potential to bind to mammarenavirus genome.

**New World Mammarenaviruses**					
**Clade A Viruses**	**Total miRNAs (Binding-Sites)**		**Clade B Viruses**	**Total miRNAs (Binding-Sites)**	
	**L Segment**	**S Segment**		**L Segment**	**S Segment**
Allpahuayo virus	12	04	Amapari virus	14	03
Bear Canyon virus	10	05	Aporé virus	15(16)	06
Flexal virus	11	03	Chapare virus	15	04
Paraná virus	09	07	Cupixi virus	23(25)	05
Pichindé virus	14	04	Guanarito virus	12	07
Pirital virus	13	09	Junín virus	13	05
Tamiami virus	12(13)	03(04)	Machupo virus	17	08
Whitewater Arroyo virus	22(23)	06	Sabiá virus	09	06(07)
**Total binding-sites**	**105**	**42**	Tacaribe virus	14	02
**Clade C viruses**	**Total microRNAs**		Xapuri virus *	07	05
	**L segment**	**S segment**	**Total binding-sites**	**140**	**54**
Latino virus	16	03	* Didactically classified here as belonging to Clade B NW mammarenaviruses.		
Oliveros virus	16	10			
**Total**	**32**	**13**			
**Old World Mammarenaviruses**					
**Viruses**	**Total miRNAs (Binding-Sites)**		**Viruses**	**Total miRNAs (Binding-Sites)**	
	**L Segment**	**S Segment**		**L Segment**	**S Segment**
Alxa virus	08	12	Mariental virus	12	03
Gairo virus	11	12	Merino Walk virus	14	04
Ippy virus	17	05	Mobala virus	12	08
Lassa virus	16	08	Mopeia virus	14	04
Lijiang virus	12(13)	07	Okahandja virus	09	09(10)
Loie River virus	07	01	Ryukyu virus	19(21)	03
Lujo virus	20	06	Solwezi virus	010	04
Luna virus	13	08	Souris virus	05	09
Lunk virus	18(22)	10	Wenzhou virus	09	06
Lymphocytic choriomeningitis virus	11	05	**Total binding-sites**	**244**	**125**

**Table 2:** List of top 10 miRNAs binding to different regions of same the RNA segment of different mammarenavirus species.

miRNAs	Mammarenaviruses (Genomic Position)
**Clade A New World Viruses (L Segment)**	
hsa-miR-122b-3p	Flexal (2745–2755: RdRp)/Paraná (2814–2824: RdRp)
**Clade A New World Viruses (S Segment)**	
hsa-miR-2052	Pirital (954–944: GPC)/Bear Canyon (2422–2412: NP)/Pichindé (948–938: GPC)/Allpahuayo (945–955: GPC)
**Clade B New World Viruses (L Segment)**	
hsa-miR-122b-3p	Aporé (2977–2967: RdRp/7195–7185: 3′UTR)/Machupo (2920–2910: RdRp)
hsa-miR-147b-5p	Aporé (6826–6816: RdRp)/Machupo (1424–1434: RdRp)/Tacaribe (6771–6761: RdRp)
hsa-miR-3149	Machupo (3195–3185: RdRp)/Tacaribe (3173–3163: RdRp)/Xapuri (4629–4639: RdRp)
hsa-miR-12122	Amapari (3524–3534: RdRp)/Chapare (3552–3562: RdRp)/Tacaribe (3553–3563: RdRp)
**Old World Viruses (L Segment)**	
hsa-miR-3120-3p	Lujo (3497–3487: RdRp)/Lunk (3599–3589: RdRp)/Merino Walk (5697–5687: RdRp)/Mopeia (3591–3581: RdRp)
hsa-miR-3134	Alxa (5200–5190: RdRp)/Wenzhou (2378–2388: RdRp)/Lymphocytic choriomeningitis (1975–1985: RdRp)
**Old World Viruses (S Segment)**	
hsa-miR-4327	Lunk (2716–2706: NP)/Merino Walk (2721–2711: NP)/Mariental (2721–2711: NP)/Souris (2777–2767: NP)
hsa-miR-6516-3p	Lujo (1548–1538: NP)/Lijiang (1655–1645: NP)/Mariental (1673–1663: NP)
hsa-miR-6740-5p	Alxa (2834–2824: NP)/Gairo (2788–2778: NP)/Lassa (2784–2774: NP)/Lymphocytic choriomeningitis (2787–2777: S-IGR)

RdRp: RNA-dependent RNA polymerase; GPC: glycoprotein precursor; NP: nucleoprotein; S-IGR: noncoding intergenic region of S segment; 3′UTR: untranslated region.

**Table 3:** List of top 10 miRNAs binding to small (S) and large (L) segments of different mammarenaviruses.

miRNAs	Species (Genomic Position)	
	L Segment	S Segment
hsa-miR-122b-3p	Aporé (2977–2967: RdRp/7195–7185: 3′UTR)	Alxa (1328–1338: GPC)
	Flexal (2745–2755: RdRp)	
	Machupo (2920–2910: RdRp)	
	Paraná (2814–2824: RdRp)	
hsa-miR-9-5p	Pichindé (4923–4933: RdRp)	Tacaribe (2639–2649: NP)
	Ryukyu (4996–5006: RdRp)	
	Mobala (5050–5060: RdRp)	
hsa-miR-3611	Aporé (3887–3897: RdRp)	Xapuri (1849–1859: NP)
	Flexal (3774–3784: RdRp)	
hsa-miR-3617-5p	Pichindé (7003–6993: RdRp)	Mopeia (2392–2402: NP)
		Paraná (1198–1208: GPC))
hsa-miR-1229-3p	Cupixi (4426–4436: RdRp)	Lassa (2783–2793: NP)
	Oliveros (6625–6635: RdRp)	
hsa-miR-3085-5p	Amapari (3334–3324: RdRp)	Lujo (1316–1306: GPC)
	Lunk (1709–1699: RdRp)	
hsa-miR-4256	Tamiami (1844–1834: RdRp)	Chapare (856–866: GPC)
	Xapuri (2518–2528: RdRp)	
hsa-miR-3913-5p	Chapare (2959–2969: RdRp)	Luna (2453–2463: NP)
	Lujo (2961–2971: RdRp)	
hsa-miR-4735-3p	Lassa (2553–2563: RdRp)	Whitewater Arroyo (1734–1744: NP)
	Merino Walk (2369–2359: RdRp)	
hsa-miR-4762-5p	Chapare (1305–1315: RdRp)	Paraná (2680–2870: NP)
	Latino (3939–3949: RdRp)	

RdRp: RNA-dependent RNA polymerase; GPC: glycoprotein precursor; NP: nucleoprotein; 3′UTR: untranslated region.

**Table 4:** MicroRNAs biding to different regions of the same mammarenavirus genomic segment.

microRNAs	Mammarenaviruses	Genomic Position
**Clade A (L Segment)**		
hsa-miR-6083	Whitewater Arroyo	1597–1607: RdRp/5470–5480: RdRp
hsa-miR-7856-5p	Tamiami	3946–3956: RdRp/4552–4562: RdRp
**Clade B (L Segment)**		
hsa-miR-122b-3p	Aporé	2977–2967: RdRp/7195–7185: 3′UTR
hsa-miR-376a-3p	Cupixi	1543–1553: RdRp/3120–3130: RdRp
hsa-miR-376b-3p	Cupixi	1543–1553: RdRp/3120–3130: RdRp
**Clade B (S Segment)**		
hsa-miR-5700	Sabiá	1039–1029: GPC/1030–1040: GPC
**Old World (L Segment)**		
hsa-miR-4460	Lijiang	209–199: Z/4747–4737: RdRp
hsa-miR-8485	Ryukyu	354–364/356–366/388–398 (L-IGR)
hsa-miR-8485	Lunk	418–428/420–430/422–432/424–434/426–436 (L-IGR)

RdRp: RNA-dependent RNA polymerase; GPC: glycoprotein precursor; Z: Z protein; L-IGR: noncoding intergenic region of L segment; 3′UTR: untranslated region.

## Supplementary Materials

The following are available online at https://www.mdpi.com/2073-4409/9/11/2525/s1, Figure S1: List of all 75 pathways found, Figure S2: All pathways found for each clade, Figure S3: All 69 pathways with at least one experimentally validated interactions for miRNAs, Figure S4: All 75 pathways distributed according to amount of microRNA interactions for each virus, Table S1: All microRNAs found, their binding sites and the location on the viral genome, Table S2: List of all miRNAs binding to different regions of same the RNA segment of different mammarenavirus species, Table S3: List of all miRNAs binding to S and L segments of different mammarenaviruses.
