# Supplementary material for: MicroRNAs and Mammarenaviruses: Modulating Cellular Metabolism
Source: Cells. 2020 Nov 23;9(11):2525. doi: 10.3390/cells9112525 (PMC7709035; doi:10.3390/cells9112525)
Supplement: Supplementary file 1 [file cells-09-02525-s001.zip › cells-981865-supplementary/Supplementary/Supplementary table 2.pdf]

Table 2. List of all miRNAs binding to different regions of same the RNA segment of different mammarenavirus species.

microRNAs	Species (Genomic position)	
**New World – Clade A (L segment)**		
hsa-miR-122b-3p	Flexal (2745 – 2755)	Paraná (2814 – 1824)
hsa-miR-181b-2-3p	Allpahuayo (541 – 531)	Flexal (4887 – 4877)
hsa-miR-548aa	Allpahuayo (5822 – 5832)	Whitewater Arroyo (6214 – 6224)
hsa-miR-548ap-3p	Allpahuayo (5822 – 5832)	Whitewater Arroyo (6214 – 6224)
hsa-miR-548d-3p	Allpahuayo (5821 – 5831)	Whitewater Arroyo (6213 – 6223)
hsa-miR-548t-3p	Allpahuayo (5822 – 5832)	Whitewater Arroyo (6214 – 6224)
hsa-miR-4299	Allpahuayo (3476 – 3486)	Tamiami (3519 – 3529)
**New World – Clade A (S segment)**		
hsa-miR-2052	Pirital (954–944)	Bear Canyon (2422 – 2412)
	Pichindé (948–938)	Allpahuayo (945 – 955)
hsa-miR-5696	Allpahuayo (1239–1249)	Flexal (1243 – 1253)
hsa-miR-6830-3p	Pirital (1328–1338)	Pichindé (1322 – 1332)
**New World – Clade B (L segment)**		
hsa-miR-122b-3p	Aporé (2977–2967/7195 – 7185)	Machupo (2920–2910)
hsa-miR-147b-5p	Aporé (6826–6816)	Machupo (1424–1434)
	Tacaribe (6771 – 6761)	
hsa-miR-545-3p	Sabiá (560–550)	Aporé (628 – 618)
hsa-miR-620	Amapari (449–439)	Junín (4114 – 4124)
hsa-miR-1200	Cupixi (124–134)	Guanarito (665–675)
hsa-miR-1262	Machupo (2451 – 2441)	Tacaribe (2432–2422)
hsa-miR-3149	Machupo (3195 – 3185)	Tacaribe (3173–3163)
	Xapuri (4629 – 4639)	
hsa-miR-4271	Chapare (2908–2898)	Tacaribe (2909 – 2899)
hsa-miR-4291	Sabiá (1745–1735)	Guanarito (5106 – 5116)
hsa-miR-12122	Amapari (3524–3534)	Chapare (3552 – 3562)
	Tacaribe (3553 – 3563)	
**New World – Clade B (S segment)**		
hsa-miR-320a-5p	Junín (2412–2422)	Guanarito (2385 – 2395)
hsa-miR-329-5p	Chapare (2034–2044)	Guanarito (2015 – 2025)
**Old World (L segment)**		
hsa-miR-9-5p	Ryukyu (4996 – 5006)	Mobala (5050–5060)

hsa-miR-181b-5p	Ippy (1618–1608)	Luna (3565 – 3555)
hsa-miR-181d-5p	Ippy (1618–1608)	Luna (3565–3555)
hsa-miR-3120-3p	Lujo (3497–3487)	Lunk (3599–3589)
	Merino Walk (5697 – 5687)	Mopeia (3591–3581)
hsa-miR-3134	Alxa (5200 – 5190)	Wenzhou (2378–2388)
	Lymphocytic choriomeningitis (1975–1985)	
hsa-miR-3189-5p	Lijiang (255–265)	Mopeia (284–294)
hsa-miR-3941	Ryukyu (1225–1215)	Lymphocytic choriomeningitis (896–906)
hsa-miR-4282	Okahandja (3742–3732)	Loie River (2784–2794)
hsa-miR-4460	Lassa (209–199)	Lijiang (209–199/4747–4737)
hsa-miR-4465	Mopeia (1034–1044)	Ryukyu (1911–1921)
hsa-miR-4735-3p	Lassa (2553–2563)	Merino Walk (2369–2359)
hsa-miR-4753-5p	Lunk (618–608)	Lujo (5150–5160)
hsa-miR-4760-5p	Solwezi (493–483)	Lunk (575 – 565)
hsa-miR-4780	Ippy (670–680)	Lunk (864–854)
hsa-miR-5681a	Luna (1709–1699)	Mariental (1649–1639)
hsa-miR-5586-3p	Solwezi (6939–6949)	Mopeia (7070 – 7080)
hsa-miR-6806-3p	Alxa (6870–6880)	Lujo (131–121)
hsa-miR-6859-5p	Lassa (1706–1696)	Solwezi (2070–2060)
hsa-miR-7109-5p	Lijiang (451–441)	Ippy (602–612)
hsa-miR-8485	Ryukyu (354–364/356–366/388–398)	Lunk (418 – 428/420–430 / 422 – 432 / 424 – 434 / 426 – 436)
hsa-miR-9903	Okahandja (1879 – 1889)	Luna (1983 – 1993)
**Old World (S segment)**		
hsa-miR-1245b-3p	Lijiang (2975–2965)	Mobala (2983–2973)
hsa-miR-1267	Lijiang (2069–2059)	Ryukyu (2124–2114)
hsa-miR-3650	Souris (2438–2448)	Wenzhou (2383–2393)
hsa-miR-3672	Ippy (1215–1225)	Lunk (1217–1227)
hsa-miR-4327	Lunk (2716–2706)	MerinoWalk (2721–2711)
	Mariental (2721–2711)	Souris (2777–2767)
hsa-miR-4773	Ryukyu (1893–1883)	Mopeia (1906–1896)
hsa-miR-5696	Alxa (1242–1232)	Lymphocyticchoriomeningitis (1242–1232)

hsa-miR-6516-3p	Lujo (1548 – 1538)	Lijiang (1655 – 1645)
	Mariental (1673 – 1663)	
hsa-miR-6740-5p	Alxa (2834 – 2824)	Gairo (2788 – 2778)
	Lassa (2784 – 2774)	Lymphocytic choriomeningitis (2787 – 2777)
