# Supplementary material for: MicroRNAs and Mammarenaviruses: Modulating Cellular Metabolism
Source: Cells. 2020 Nov 23;9(11):2525. doi: 10.3390/cells9112525 (PMC7709035; doi:10.3390/cells9112525)
Supplement: Supplementary file 1 [file cells-09-02525-s001.zip › cells-981865-supplementary/Supplementary/Supplementary table 3.pdf]

Table3. ListofallmiRNAsbindingtoSandLsegmentsofdifferentmammarenaviruses.

microRNAs	Species(Genomicposition)	
	Lsegment	Ssegment
hsa-miR-122b-3p	Aporé(2977–2967/7195–7185)	Alxa(1328–1338)
	Flexal(2745–2755)	
	Machupo(2920–2910)	
	Paraná(2814–2824)	
hsa-miR-9-5p	Pichindé(4923–4933)	Tacaribe(2639–2649)
	Ryukyu(4996–5006)	
	Mobala(5050–5060)	
hsa-miR-3611	Aporé(3887–3897)	Xapuri(1849–1859)
	Flexal(3774–3784)	
hsa-miR-3617-5p	Pichindé(7003–6993)	Mopeia(2392–2402)
		Paraná(1198–1208)
hsa-miR-1229-3p	Cupixi(4426–4436)	Lassa(2783–2793)
	Oliveros(6625–6635)	
hsa-miR-3085-5p	Amapari(3334–3324)	Lujo(1316–1306)
	Lunk(1709–1699)	
hsa-miR-4256	Tamiami(1844–1834)	Chapare(856–866)
	Xapuri(2518–2528)	
hsa-miR-3913-5p	Chapare(2959–2969)	Luna(2453–2463)
	Lujo(2961–2971)	
hsa-miR-4735-3p	Lassa(2553–2563)	WhitewaterArroyo(1734–1744)
	MerinoWalk(2369–2359)	
hsa-miR-4762-5p	Chapare(1305–1315)	Paraná(2680–2870)
	Latino(3939–3949)	
hsa-miR-4667-5p	Solwezi(2213–2203)	Sabiá(1568–1578)
		Xapuri(1686–1676)
hsa-miR-5700	Sabiá(1039–1029/1030–1040)	Okahandja(1084–1074/1075–1085)
hsa-miR-376a-3p	Cupixi(1543–1553/3120–3130)	Paraná(66–76)
hsa-miR-376b-3p	Cupixi(1543–1553/3120–3130)	Paraná(66–76)
hsa-miR-542-3p	Xapuri(6128–6138)	Amapari(235–225)
hsa-miR-4251	Pichindé(742–732)	MerinoWalk(2239–2229)
hsa-miR-3167	LoieRiver(1277–1267)	WhitewaterArroyo(2804–2814)
hsa-miR-3646	Lijiang(1865–1855)	Machupo(896–906)
hsa-miR-3529-3p	Guanarito(4043–4033)	Alxa(3051–3041)
hsa-miR-4326	Aporé(4447–4457)	Gairo(171–181)
hsa-miR-6764-5p	Flexal(4707–4717)	Machupo(2867–2877)
hsa-miR-548b-3p	Amapari(4037–4047)	Lassa(2305–2295)
hsa-miR-4699-5p	Lassa(5320–5310)	Chapare(761–771)
hsa-miR-301b-3p	Pichindé(755–765)	Lassa(100–90)
hsa-miR-3130-5p	Lassa(2713–2723)	Pichindé(1544–1534)

hsa-miR-1255b-2-3p	Allpahuayo(6270–6280)	Latino(2001–1991)
hsa-miR-8087	Pirital(5640–5630)	Wenzhou(792–802)
hsa-miR-6844	Amapari(2980–2970)	MerinoWalk(1740–1750)
hsa-miR-10393-3p	Machupo(4049–4059)	Sabiá(1802–1792)
hsa-miR-4503	WhitewaterArroyo(3624–3634)	Luna(2002–1992)
hsa-miR-130b-3p	Pichindé(755–765)	Lassa(100–90)
hsa-miR-330-5p	Gairo(2305–2315)	Machupo(2704–2694)
hsa-miR-335-5p	Junín(1420–1410)	Lassa(633–623)
hsa-miR-7114-5p	Alxa(458–448)	WhitewaterArroyo(503–493)
