# Supplementary figures and images for: MicroRNAs and Mammarenaviruses: Modulating Cellular Metabolism
Source: Cells. 2020 Nov 23;9(11):2525. doi: 10.3390/cells9112525 (PMC7709035; doi:10.3390/cells9112525)

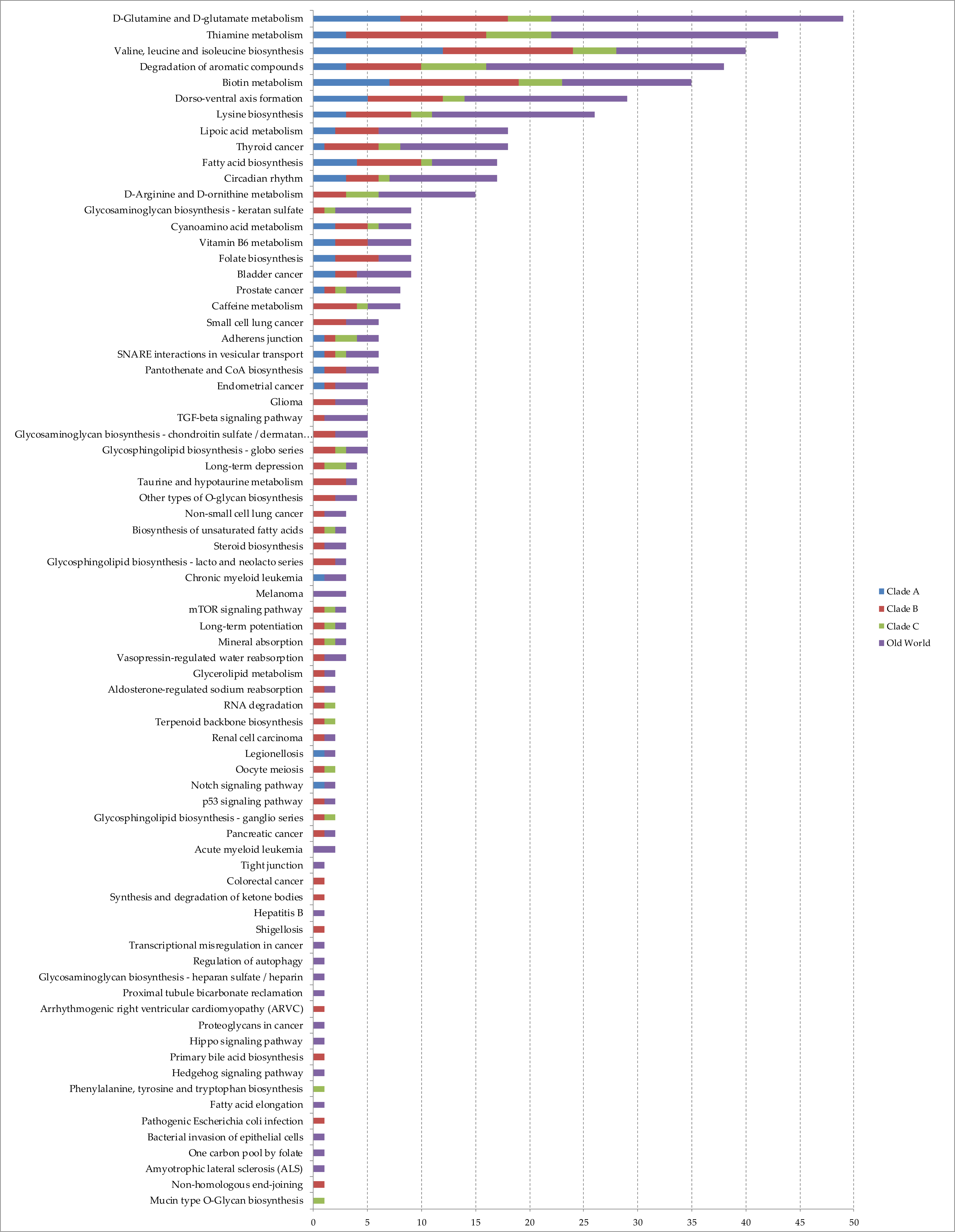

Supplement: Supplementary file 1 [file cells-09-02525-s001.zip › cells-981865-supplementary/Supplementary/Supplementary figure 1.tiff]

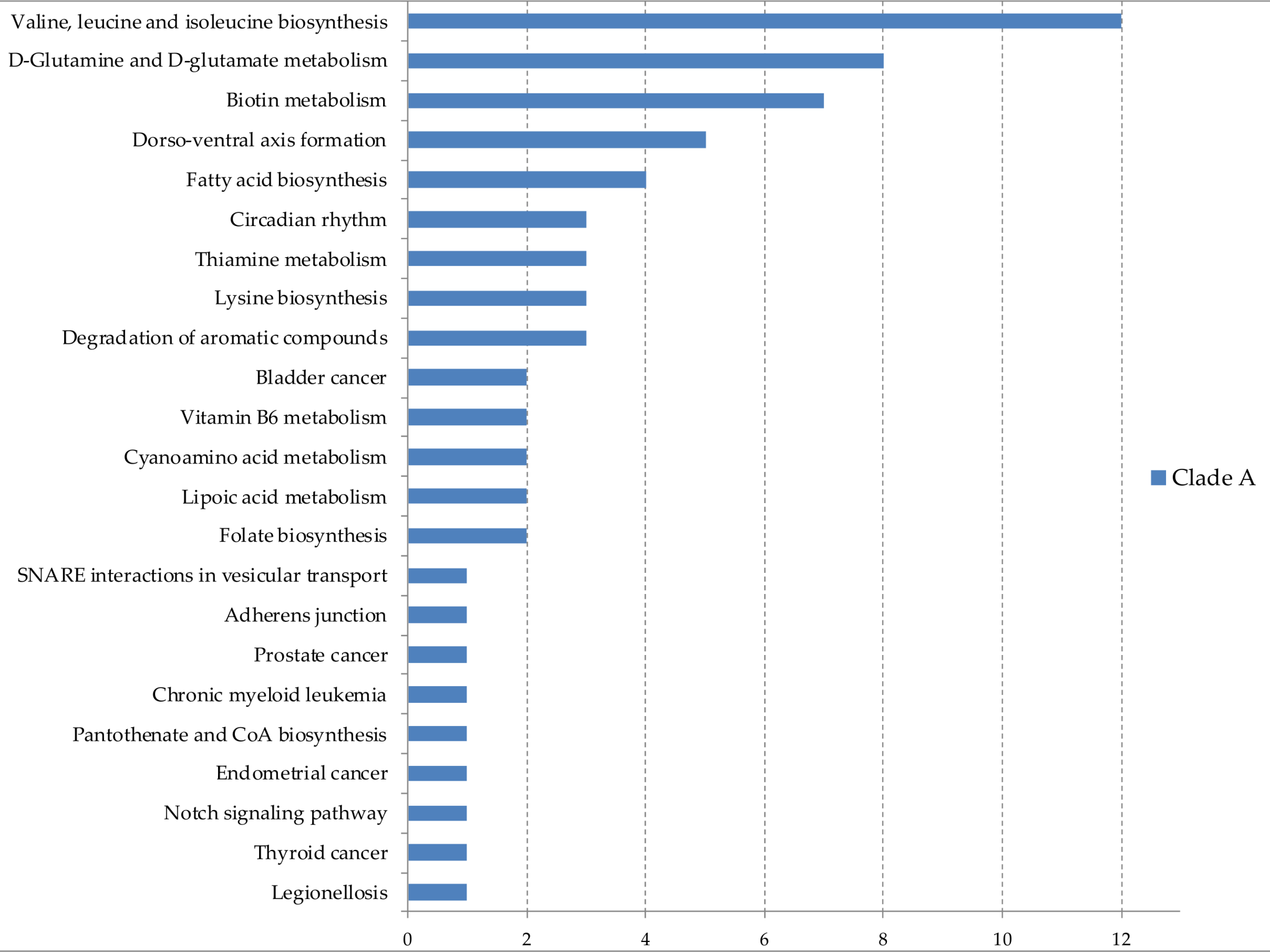

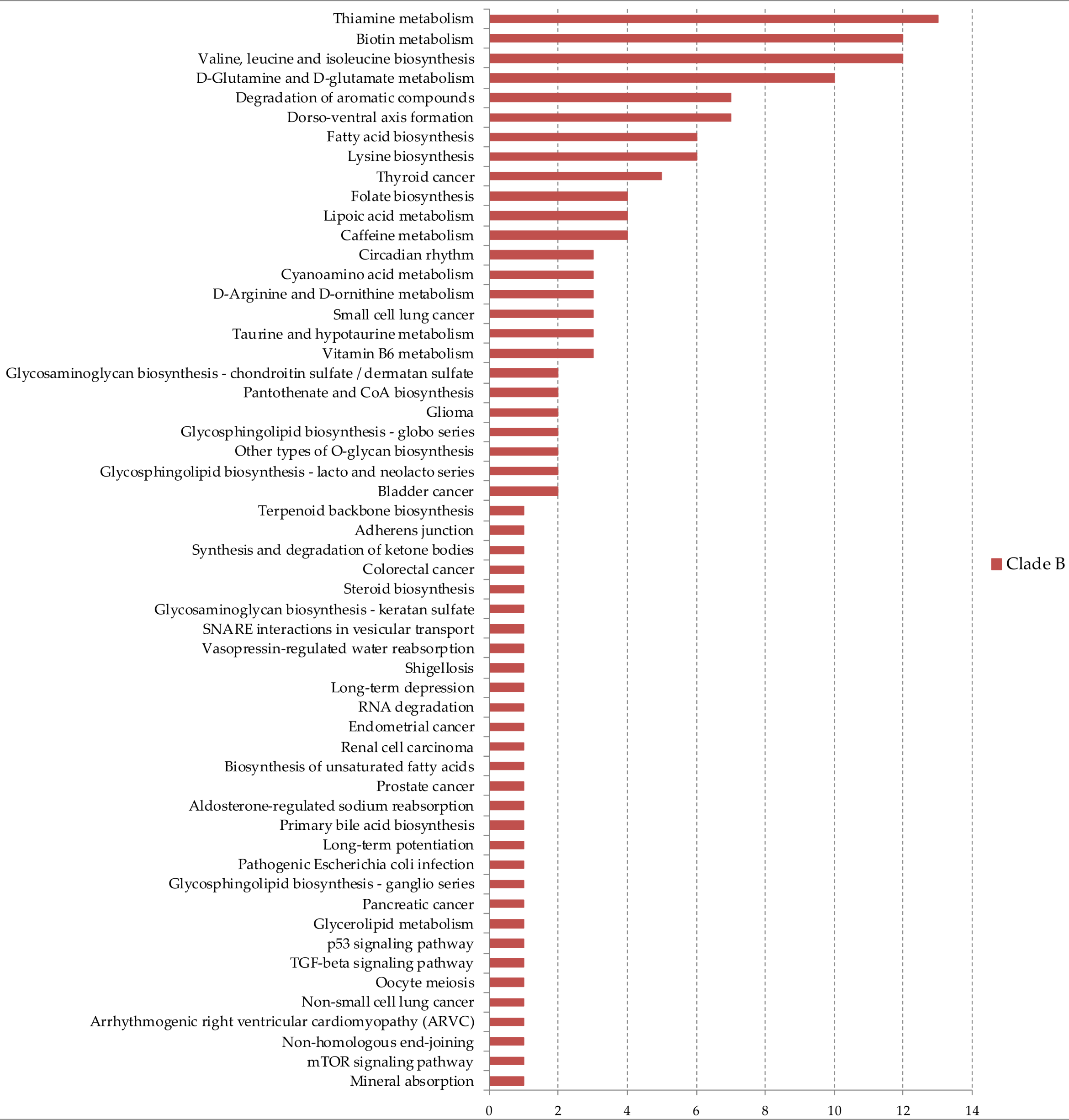

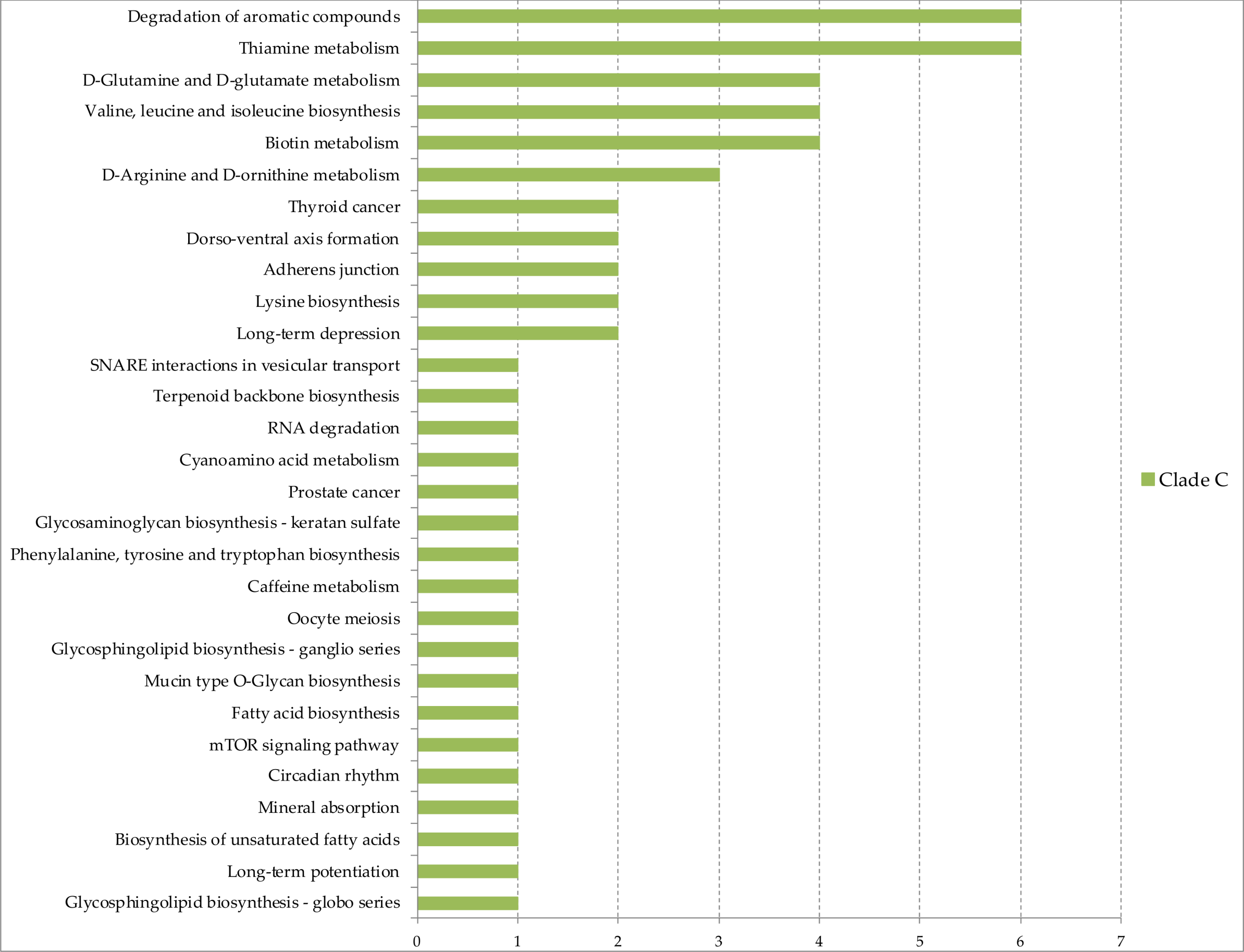

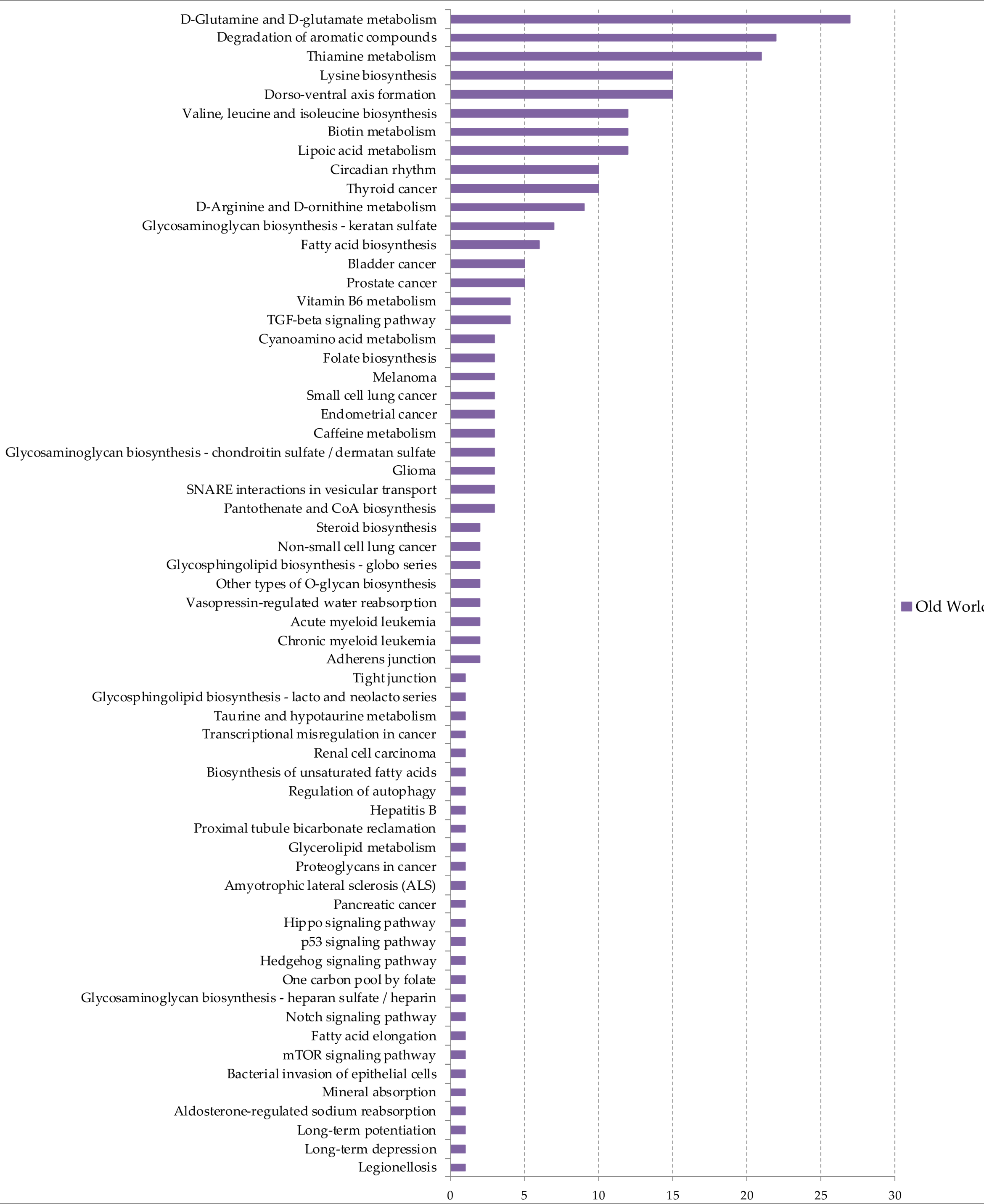

Supplement: Supplementary file 1 [file cells-09-02525-s001.zip › cells-981865-supplementary/Supplementary/Supplementary figure 2.pdf]

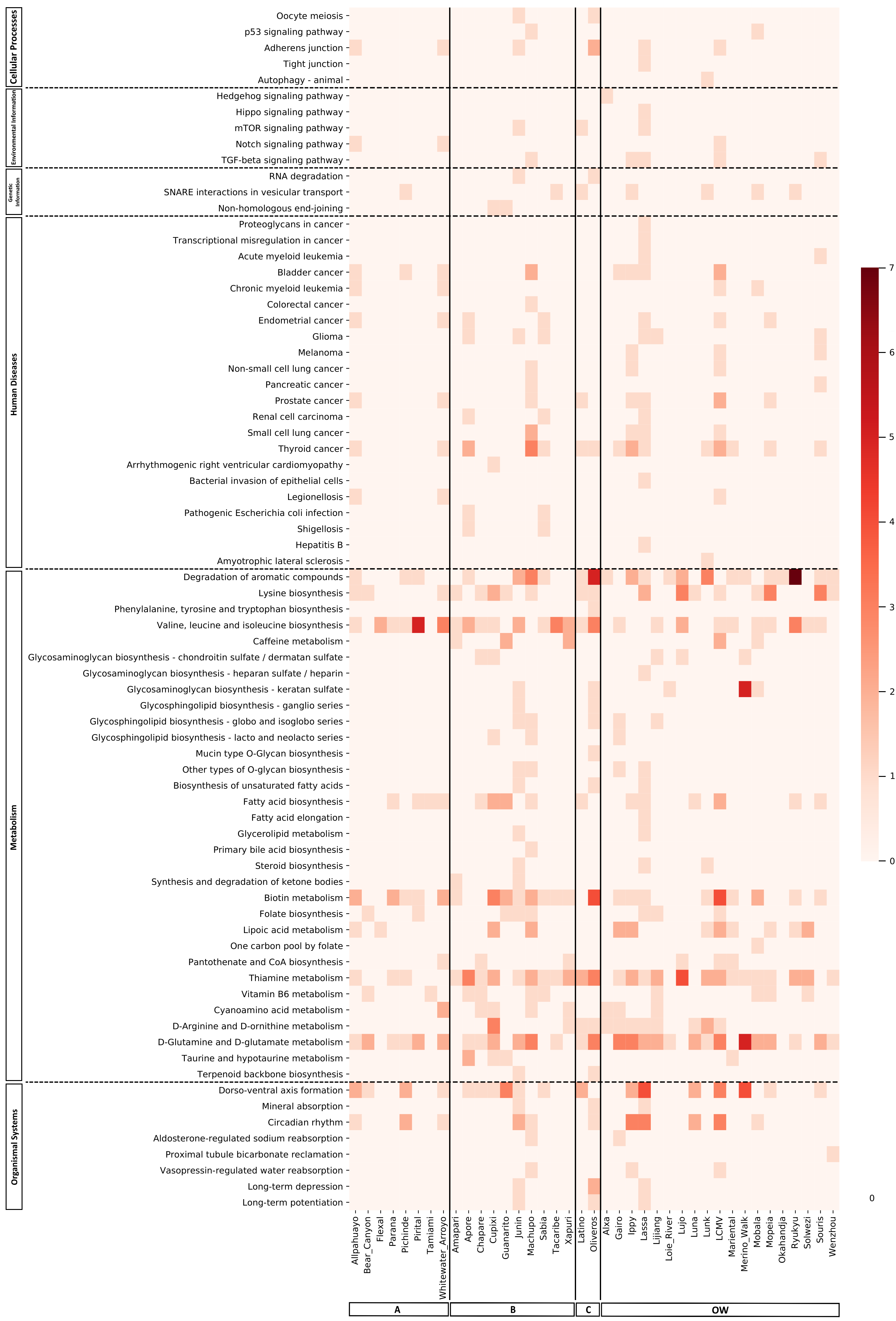

Supplement: Supplementary file 1 [file cells-09-02525-s001.zip › cells-981865-supplementary/Supplementary/Supplementary figure 4.tiff]
